# Production, consumption, and market supply of edible crickets: insights from East Africa

**DOI:** 10.1186/s40100-023-00272-9

**Published:** 2023-08-09

**Authors:** Arnold L. Musungu, Beatrice W. Muriithi, Changeh J. Ghemoh, Dorothy Nakimbugwe, Chrysantus M. Tanga

**Affiliations:** 1 Center for Development Research (ZEF), University of Bonn, Genscherallee Str. 3, 53113 Bonn, Germany; 2 International Centre of Insect Physiology and Ecology (Icipe), P.O. Box 30772-00100, Nairobi, Kenya; 3 Centre for African Bio-Entrepreneurship (CABE), P.O. Box 25535-00603, Lavington, Nairobi, Kenya; 4 Department of Food Technology and Nutrition, School of Food Technology, Nutrition, and Bioengineering, Makerere University, P.O. Box 7062, Kampala, Uganda

**Keywords:** Cricket production, Alternative protein, Kenya, East Africa

## Abstract

Globally, crickets are gaining recognition as a valuable alternative protein source for human consumption due to their lower resource requirement and ecological footprint compared to traditional livestock. In this paper, we examine strategies that may expedite the sustainable domestication of crickets as a food source. Using survey data from 306 households in western Kenya, we find that supplying cricket production starter kits, granting access to credit facilities, encouraging participation in farmer groups, and fostering partnerships can enhance the adoption of cricket farming. Moreover, we provide new evidence that institutional training significantly increases cricket yields while embracing cricket consumption (i.e. entomophagy) increases market supply. These findings underscore the importance of technical training, provision of production starter kits, and raising awareness about entomophagy to achieve sustainable mass production and adoption of cricket farming.

## Introduction

The United Nations (UN) projects a global population growth of approximately 9.5 billion by 2050, which will result in an unprecedented increase in the global demand for food (UN 2017). Precisely, it will require twice the current food production to feed the world by 2050 (FAO 2009). In addition, with increasing globalization, urbanization, and expected growth in household incomes and global affluence, resource economists anticipate a significant increase in per capita consumption of meat and other forms of animal proteins. These projections have cast doubt on the potential of livestock as a sustainable source of protein, whose demand is estimated to swell to 455 million tons (70% increase) per year by 2050 (MacLeod et al. 2018). Besides, the meat industry is said to have the highest carbon footprint, significantly contributing to deforestation, soil degradation, water stress, and biodiversity loss (Machovina et al. 2015; Poore and Nemecek 2018; Crenna et al. 2019; Henry et al. 2019; Parlasca and Qaim 2022). This evidence taken together compellingly suggests that conventional animal protein sources, including beef, pork, and chicken, may not sustainably meet the demand, subsequently opening a window for exploring alternative and more sustainable sources.

Against this background and building on the ongoing global efforts to establish insects as an alternative food group, the International Center of Insect Physiology and Ecology (*icipe*) and other partners have over the past decade invested in research and technologies to increase awareness, acceptance, production, and consumption of insects both for food and livestock feed in sub-Saharan Africa (SSA). This collaboration has led to the discovery of a new, previously scientifically undescribed edible cricket (*Scapsipedus icipe*) with great promise for mass production for human consumption and inclusion as an alternative protein ingredient in chicken feed (Tanga et al. 2018). While cricket farming for human food and livestock feed is common in other parts of the world, its domestication is relatively new in Kenya and the rest of Africa (van Huis 2013; Lundy and Parrella 2015; Ayieko et al. 2016). Nonetheless, several studies have confirmed the demand for certain insects for human food and animal feed in Kenya (Chia et al. 2020; Alemu and Olsen 2018; Alemu et al. 2018; Alemu et al. 2015). Specifically, in a survey by Chia et al. (2020) that uses the contingent valuation approach to elicit willingness to pay, about 70% of farmers demonstrated a positive willingness to pay for insect-based livestock feed at the prevailing market prices. The demand for the insect-based feeds further responded positively to discounted prices. Additionally, using a choice experiment to elicit demand for insects as food in Kenya, Alemu and Olsen (2018) find that nutrition-sensitive consumers are willing to pay KES.275^1^ per 200-g of termite-based food product. These results, taken together, instill hope in the acceptance of crickets.

In light of this, we study the production, consumption and market supply of edible crickets in Kenya. We leverage the existing efforts by *icipe* and the Flying Food project^2^ through which rural households were invited to participate in a training program that would prepare them for domestic cricket farming. We have three objectives: Firstly, we hypothesize that sustainable mass production and market supply are dependent on institutional and household socio-economic and behavioral factors. Secondly, we test whether access to institutional training sources increases cricket yields. Lastly we analyze the effects of pre-existing household consumption behavior (cricket entomophagy^3^) on market supply of crickets by these households. We apply a distinct empirical strategy for each objective.

In the first objective, we theorize that the adoption of cricket farming constitutes a two-phase process: First, households decide whether to adopt the enterprise conditional on receiving training, and secondly, following this decision, they determine the production quantity. In this regard, we use the Heckman two-step sample selection model in our first objective. The findings of the first stage show a negative correlation between the distance to a main road (indicating remoteness) and the probability of adopting cricket farming. Conversely, factors such as group membership, household acceptance of crickets as food, and the provision of cricket production starter kits post-training are positively correlated with the likelihood of household adoption. In the second stage of the model, we find that remoteness negatively impacts yield, while access to credit appears to boost yield.

In the second research question, we examine the impact of institutional training sources on cricket production the multinomial endogenous treatment effects (METE) model. We find that access to training from NGOs and a public research university increase cricket yields by 55.4% and 70.8%, respectively. Lastly, we examine the impact of consumption behavior on market supply using an endogenous switching regression (ESR) model. The first stage ESR estimates reveal that geographical remoteness and market inaccessibility negatively correlate with the likelihood of cricket consumption. In contrast, the awareness of crickets as a nutritious food source is positively linked to consumption rates. Furthermore, collective cricket farming activities show a positive correlation with market supply among cricket-consuming households. We also observe a marginal association between higher education levels and increased market supply among non-consuming households. Estimates from the second stage of the ESR model reveal that cricket consumption boosts market supply by 20.5%. Additionally, we find suggestive evidence that if non-consuming households were to adopt cricket consumption, there is potential to augment their market supply by 29.5%.

The rest of the paper is organized as follows: We briefly present a literature review in “Literature Review” Section. “Methods” Section describes the data sources and analytical strategies while “Results and Discussion” Section presents and discusses our results. Lastly, we conclude and highlight policy implications in “Conclusions and policy implications” Section.

## Literature review

Livestock value chains are already an ecological stressor and a threat to global ecosystems. For instance, there is a growing consensus that animal-based foods cause more greenhouse gas (GHG) emissions as compared to insect-based foods (Oonincx and de Boer 2012). Besides, livestock production systems require a significant amount of natural resources, such as continuous feed production, which is responsible for about 14.5% of total anthropogenic GHG emissions (Gerber et al. 2013). The ultimate environmental cost is the conversion of natural ecosystems such as forests, wetlands, and grasslands into livestock production zones.

In light of these challenges, conventional livestock production is highly regarded as an unsustainable use of natural resources. Consequently, it is unlikely to meet the projected future protein demand without remarkable environmental tradeoffs, thus necessitating serious consideration for alternative protein food groups (Patel et al. 2019). These developments have seen edible insects receive considerable contemplation, as various insects are already well accepted and consumed as food in some parts of the world (Murefu et al. 2019; Zielinska et al. 2018). Besides, studies show that there is consumption (entomophagy) of more than 552 species of insects by some 300 million people in 45 African countries alone. Globally, over 2000 species are consumed as delicacies (Yen 2009) by at least 2 billion people in parts of Asia, Africa, and South America (Van Huis et al. 2013a, b; Jongema 2015; Kelemu et al. 2015). Among these edible insects, beetles are the most commonly consumed (31%), followed by caterpillars (18%), bees, wasps, and ants (14%), grasshoppers, locusts, and crickets (13%), cicadas, leafhoppers, planthoppers, scale insects, and true bugs (10%), termites (3%), dragonflies (3), flies (2%), and others (5%) (Van Huis et al. 2013a, b). In the EU region, crickets are reported to have a higher potential for applications in the food industry (Van der Spiegel et al. 2013). This recent scrutiny of edible insects is part of composite strategies for providing alternative food sources while achieving global nutrition security (van Huis 2015).

The growing interest in insects as an alternative food group is mainly because of their nutritional, economic, and environmental values. Regarding nutritional and economic values, edible insects are rich in protein and have outstanding production efficiency (Kohler et al. 2019; Nongonierma and FitzGerald 2017). Additionally, following Oonicx and de Boer (2012), it is presumed that insect production has very little impact on the natural environment compared to conventional meat production. Notably, it is argued that insect production has few requirements in terms of land and water and has relatively low GHG emissions compared to livestock production systems. Besides, most of the insect body weight is consumed and digested (80%), compared to chicken (55%), and cattle (40%) (WRAP 2017).

Despite their massive potential for food and animal feeds and compelling evidence on the acceptance of selected insects such as black soldier flies and termites, household insect farming (domestication) in Kenya is not well established, and consumption is likely constrained by various factors. Firstly, most insects are still harvested from their natural environments (usually forests), implying that their supply is dependent on location and seasonality (Nonaka 2009). Secondly, there are food safety concerns with insect collection, handling, and consumption, which calls for more rigorous research (Belluco et al. 2013). Thirdly, insect foods are still in the transition stage, so optimal consumption depends on technologies that assimilate other widely accepted foods (Kohler et al. 2019; Patel et al. 2019). These gaps in the literature motivate the hypotheses we test in this study.

## Methods

### Data sources

**Fig. 1:**
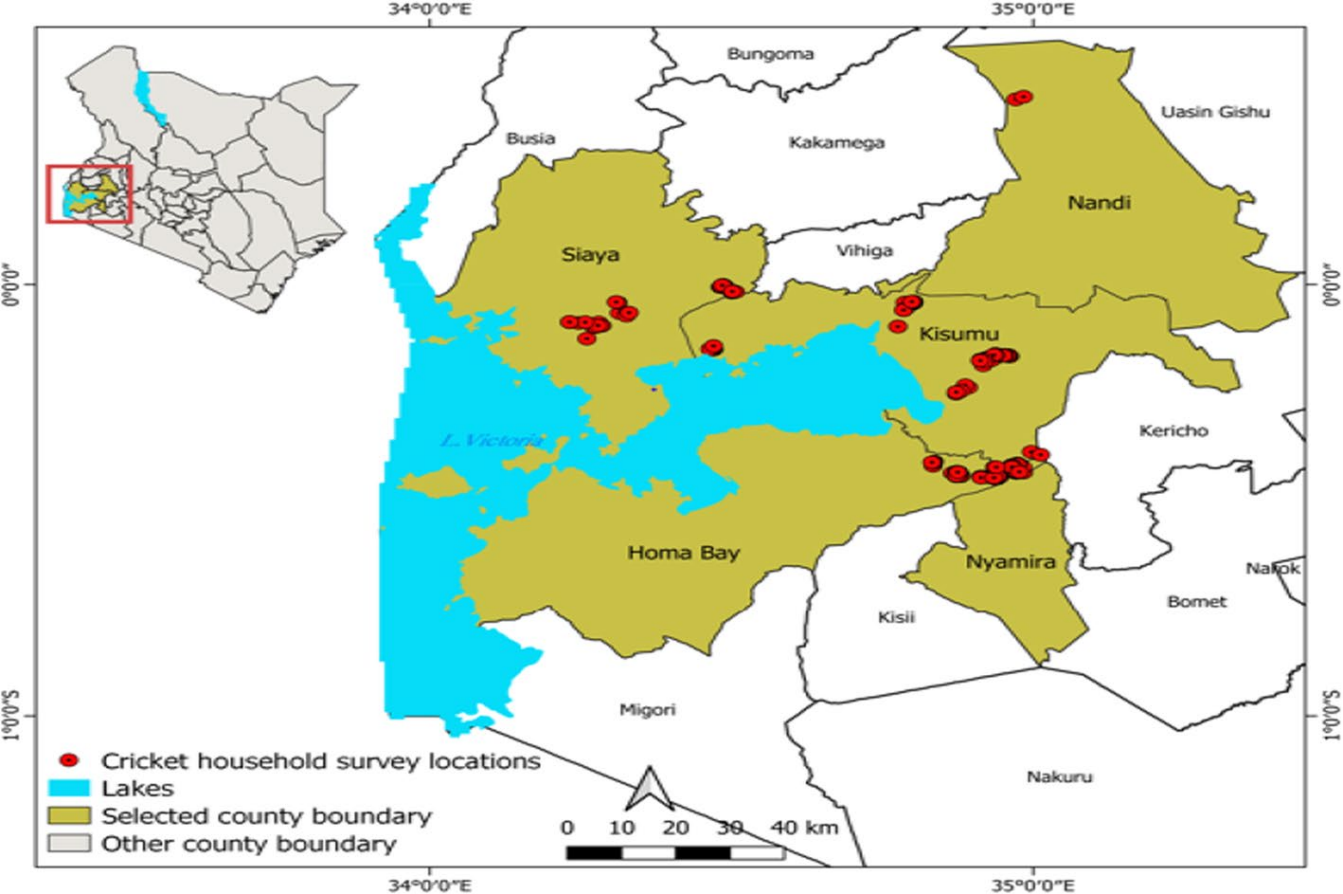
Map of the study sites

We conducted household surveys across three counties in Western Kenya: Kisumu, Siaya, and Homabay (refer to Fig. 1). These counties served as the benchmark sites for the Flying Food project in 2013, an initiative aimed at establishing small-scale cricket rearing stations on household farms. This project was a collaborative effort involving research institutions, local public universities, and a non-governmental organization (NGO), providing training on cricket rearing to interested farmers. These farmers were anticipated to adopt cricket farming following their training.

Our data collection employed both purposive and snowball sampling techniques. The former was instrumental in identifying individuals across the three counties who had participated in any Flying Food training sessions.^4^ During our preliminary visits to the study sites, it became evident that a considerable number of farmers, who had not originally participated in the Flying Food Project’s training sessions, demonstrated interest and adopted cricket rearing by learning from their fellow farmers. This observation guided our decision to incorporate snowball sampling into our study design, enabling us to reach these farmers. Our chosen sampling methods resulted in a sample size of 306 households. However, after excluding units with missing data on crucial variables, our analysis sample is 301 households.

The data collection exercise was conducted by well-trained enumerators supervised by *icipe*’s researchers, using a structured and pretested questionnaire programmed in a computer-assisted personal interviews (CAPI) survey tool. The questionnaire contained different modules that captured detailed information on household socioeconomic characteristics, awareness and perceptions on insects farming and consumption, access to cricket farming training, cricket production, marketing and associated constraints, and institutional support services.

### Empirical strategy

#### Heckman two-step sample selection model to assess cricket production decision

Domestic cricket farming being relatively new (Halloran et al. 2020), household production takes a two-step process. The first stage is the decision to adopt cricket rearing after receiving training, while the second stage is the household’s total production after adopting the venture. Since the second stage is dependent on the first stage, it is considered a sub-sample of the first stage. As a result, it is more likely that this second stage sub-sample is non-random and different from those who did not take up cricket farming after receiving training, thus sample selection bias is very likely. To correct for this possible selectivity bias, we apply Heckman’s two-step sample selection model (Heckman 1976) where we first estimate the selection equation followed by the outcome equation.

The first (selection) equation in a Heckman model is a probit estimator. We, therefore, specify our selection equation as:

1 $$Y_{i}^{*} = \beta_{0} + \beta_{ij} X_{ij} + \varepsilon_{i}$$

where $Y_{i}^{*}$ is a binary response variable with *Y*_*i *_= *1* if the respondent started practicing (adopters) cricket farming after receiving training and *Y*_*i *_= *0* if otherwise (non-adopters). *β*_0_ is the intercept, *β*_*ij*_ is a vector of parameters to be estimated; *X*_*ij*_ is a vector of explanatory variables which explain cricket farming adoption behavior; $\varepsilon_{i}$ is a standard normal distributed error term that is independent of *X*_*ij*_ and symmetrically distributed about 0.

The probit estimation also provides the value of inverse mills ratio (IMR) *λ* which is the ratio of the ordinate of a standard normal distribution to the tail area of the distribution (Greene 2003) as shown:

2 $$\lambda_{i} = \frac{{\varphi \left( {p + aX_{i} } \right)}}{{\emptyset \left( {p + aX_{i} } \right)}}$$

where $\varphi$ is the standard normal density function and $\emptyset$ is the standard normal distribution function.

According to (Heckman 1979), if the IMR (λi) is statistically insignificant, then there is no sample selection bias. Therefore, a statistically significant IMR implies that a significant difference exists between the farmers that adopted cricket farming after receiving training and those that did not. In estimating the outcome equation, this difference needs to be taken into consideration.

The second stage (outcome equation) estimated using OLS estimator and is specified as:

3 $$Y_{i} = \alpha_{0} + \alpha_{ij} X_{ij} + \alpha \lambda_{i} + \varepsilon_{i}$$

where $Y_{i}$ is our continuous outcome variable (quantity of crickets produced in kilograms), $\alpha$ are parameters to be estimated, *X*_*ij*_ is a vector of explanatory variables, $\lambda_{i}$ is the IMR from the probit estimation and $\varepsilon$ is the error term.

#### Multinomial endogenous treatment effects model to estimate the effect of training on cricket production

In this study, potential cricket farmers had two main cricket production training sources: Public university and non-governmental organizations. In addition, due to high possibility of knowledge spillovers, farmers are also likely to receive training from fellow farmers who have receieved institutional training. Nonetheless, our aim is to evaluate the effects of institutional training sources. We theorize that farmers will choose a training source that maximizes expected utility subject to socio-economic and institutional constraints. A farmer *i* will therefore choose a training source or a bundle of training sources *j*, over any other alternative source *k*, if *V*_*ij*_ > *V*_*ik*_, k ≠ j where *V*_*i*_ is the indirect utility derived from any of the training sources.

However, when the farmers are required to choose from available cricket production training sources (providers), selection bias and endogeneity problems may arise as the choice decision could be influenced by other unobservable characteristics. Failure to account for endogeneity and selection bias can either overestimate or underestimate the actual effects of the treatment variable on the outcome variable of interest. As a result, we follow Deb and Trivedi (2006) and apply a multinomial endogenous treatment effect (METE) model to estimate the effects of training sources on cricket production. We include an instrument in the choice of training source equation for a more robust identification (Deb and Trivedi 2006). Guided by existing literature (Di Falcao et al. 2011; Kassie et al. 2013; Manda et al. 2016), we include location (county of residence) as an instrument as the location of training sources in the three study counties is plausibly exogenous to farmers. As such, our assumption is that county of residence will only affect cricket yields through participation in either of the two main training programs. The results in Appendix Table 7 indeed confirm that location is significantly correlated to access to the two training sources.

**Table 1:** Conditional expectations, treatment, and heterogeneity effects

Subsamples	Predictions		Treatment effects
Consumers	(a) $E\left( {Y_{1i} {|}C = 1} \right)$	(b) $E\left( {Y_{2i} {|}C = 1} \right)$	ATT
Non-consumers	(c)$E\left( {Y_{2i} {|}C = 0} \right)$	(d) $E\left( {Y_{1i} {|}C = 0} \right)$	ATU
Heterogeneity Effects	HC_1_	HC_2_	$TH = ATT - ATU$

(a) and (d) represent observed expected cricket yields; (c) and (d) represent counterfactual expected cricket yields; Y_1i_ = cricket yield if the cricket consuming households; Y_2i_ = cricket yield if the cricket non-consuming households; ATT = the average effect of the treatment (consumption) on the treated; ATU = the average effect of the treatment (consumption) on the untreated; HC = the effect of base heterogeneity for cricket consumers (i = 1), and non-consumers (i = 2); TH = ATT-ATU, i.e., transitional heterogeneity

The METE model estimation proceeds in two stages. The first stage applies a multinomial logit which models potential cricket farmers’ adoption decisions for the two main training sources. This first stage assumes that farmers are rational and will therefore choose a training source that maximizes their indirect utility *V*_*ij*_, specified as:

4 $$V_{ij}^{*} = z_{i}{\prime} \alpha_{j} + \mathop \sum \limits_{k = 1}^{j} \delta_{jk} l_{ik} + n_{ij}$$

where *z*_*i*_ is a vector of household socioeconomic characteristics; $\alpha_{j}$ is the vector of parameters to be estimated $l_{ik}$ is the latent factor that constitutes the households’ unobserved characteristics common to the choice of the training source and outcomes (quantities of crickets harvested) and $n_{ij}$ are the independently and identically distributed error terms.

The second stage estimates the participation effects of the training sources on the natural log of quantities (in kilograms) of crickets harvested. We specify the outcome equation as:

5 $$E\left( {y_{i} |d_{i} ,x_{i} ,l_{i} } \right) = x_{i}^{\prime } \beta + \mathop \sum \limits_{j = 1}^{j} \gamma_{j} d_{ij} + \mathop \sum \limits_{j = 1}^{j} \lambda_{j} l_{ij}$$

where *y*_*i*_ is the quantity^5^ of crickets produced by farmer *i*; *x*_*i*_ represents exogenous covariates with parameter vector β. Parameters γ_*j*_ denote the treatment effects of participating in training relative to the non-participants. If the farmer’s decision to participate in training is endogenous and assuming *di*_*j*_ to be exogenous, estimates of γ*j* would be biased and inconsistent thus necessitating exogeneity tests in outcome Eq. (5). The latent factor $l_{ij}$ represents the unobserved characteristics that may bring about self-selection. The factor-loading parameters are represented by $\lambda_{j}$. The sign of the statistically significant factor (positive or negative) implies a correlation between the outcome and the treatment through unobservable characteristics, hence evidence of negative or positive self-selection. The multinomial endogenous treatment effects model takes a Gaussian (normal) distribution since the outcome variable (cricket quantities) is continuous. Equation (5) is therefore estimated through the maximum simulated likelihood approach.

#### Endogenous switching regression to estimate the effect of household cricket consumption on market supply

Estimation of cricket production and supply gains from pre-existing consumption behavior of households based on non-experimental data is not trivial because of the need to find a good counterfactual. What we cannot observe is the production effects of those households that are already cricket consumers should they have chosen not to be cricket consumers. Neither can we observe the effects on non-consumers had they been consumers. Experimental and quasi-experimental studies would effectively address this problem by having two non-consuming insect groups to begin with: treatment and control. With this approach, the treatment group would receive adequate training on how and why they should eat crickets including how to prepare the crickets as food and the benefits of consuming insects in general (entomophagy). We would then presume that the outcomes observed on the control are statistically representative of what would have occurred without adoption.

However, routine cricket consumption is not well established in Kenya and therefore not randomly distributed into two well-defined groups of the consuming and non-consuming households, but rather the households themselves decide to consume or not to consume based on their awareness and perceptions towards crickets as an alternative food. As such, there may exist systematic differences between consumers and non-consumers due to observable household characteristics such as education, household size, access to information, income levels, etc. Further, unobservable characteristics such as attitudes, skills, and individual motivations may influence cricket consumption decisions and subsequently affect potential yields and market supply by the households (Abdulai and Huffman 2014). Therefore, in the absence of an experimental design, the selected impact estimation technique should be vital in either eliminating the aforementioned selection bias or good enough to correct for it (Khandker et al. 2009; Palmer-Jones 2010; Wooldridge 2015).

In light of the above-mentioned issues, we follow Maddala and Nelson (1975) to estimate endogenous switching regression (ESR) as it accounts for both endogeneity and sample selection bias. Some of the recent applications of the ESR in settings with endogenous treatment variable and sample selection bias include Paltasingh and Goyari (2018), Khonje et al. (2018), Kanburi et al. (2019) and Kumar et al. (2020). We proceed with the ESR model, first, by estimating a probit regression that specifies two regimes of cricket farmers: those who consume the crickets (regime 1) and the non-consumers (regime 2). The two regimes are specified from the households who adopted cricket farming after receiving training since they represent the sub-sample with observations on the outcome variable. As a result, the sub-sample for this analysis is 139. The model specification for each regime is as follows:

6a $${\text{Regime}}\,\,\,{1}:\quad Y_{1i} = \beta_{1} X_{i} + \varepsilon_{1i} if C_{i} = 1$$

6b $${\text{Regime}}\,\,\,{2}:\quad Y_{2i} = \beta_{2} X_{i} + \varepsilon_{2i} if C_{i} = 0$$

where $Y_{1i}$ and $Y_{2i}$ are quantities of crickets sold for cricket consuming and non-consuming households respectively, *X*_*i*_ represents a vector of exogenous variables thought to influence cricket rearing. The error terms are assumed to have a trivariate normal distribution, with mean vector zero and covariance matrix specified as:

7 $$Cov(\varepsilon_{1i} ,\,\,\varepsilon_{2i} ,\,\,\mu_{i} ) = \left[ {\begin{array}{*{20}c} {\sigma_{\mu }^{2} } & . & . \\ {\sigma_{\varepsilon 1\mu }^{2} } & {\sigma_{\varepsilon 1}^{2} } & . \\ {\sigma_{\varepsilon 2\mu }^{2} } & . & {\sigma_{\epsilon 2}^{2} } \\ \end{array} } \right]$$

where $\sigma_{\mu }^{2}$ is the variance of the error term in the selection equation, $\sigma_{\varepsilon 1}^{2}$ and $\sigma_{\epsilon 2}^{2}$ are variances of the error terms in the continuous equation $\sigma_{\varepsilon 1\mu }^{2}$ and $\sigma_{\varepsilon 2\mu }^{2}$ are covariance of *u*_*i*_ and ε_1i_ and ε_2i_ respectively. The structure of the error terms in Eq. (7) indicates that the error terms of the outcome equation and the error term of the selection equation are correlated which results in a non-zero expected value of ε_1i_ and ε_2i_ given *u*_*i*_ (Abdulai and Huffman 2014). Therefore, the expected values of the truncated error terms (ε_1_|*C* = 1) and *E*(ε_2_ |*C* = 0) are given as shown:

8a $$E\left( {\varepsilon_{1} {|}C = 1} \right) = E\left( {\varepsilon_{1} {|}\mu > - Z\alpha } \right) = \sigma_{\varepsilon 1\mu } \frac{{\varphi \left( {\frac{Z\alpha }{\sigma }} \right)}}{{\Phi \left( {\frac{Z\alpha }{\sigma }} \right)}} \equiv \sigma_{\varepsilon 1\mu } \lambda_{1}$$

8b $$E\left( {\varepsilon_{1} {|}C = 0} \right) = E\left( {\varepsilon_{2} {|}\mu > - Z\alpha } \right) = \sigma_{\varepsilon 2\mu } \frac{{\varphi \left( {\frac{Z\alpha }{\sigma }} \right)}}{{\Phi \left( {\frac{Z\alpha }{\sigma }} \right)}} \equiv \sigma_{\varepsilon 2\mu } \lambda_{2}$$

where φ and Φ are the standard normal probability density and cumulative distribution functions respectively. The ratio of φ and Φ evaluated at Zα is referred to as the inverse Mills ratio λ_1_ and λ_2_ which represent the selectivity terms. If the estimated covariance $\sigma_{\varepsilon 1}^{2}$ and $\sigma_{\epsilon 2}^{2}$ are statistically significant, then the decision to consume crickets and the outcome variable (quantities harvested) are correlated. This confirms the presence of a sample selectivity bias thus justifying the application of ESR (Maddala and Nelson 1975).

While the model parameters can be identified through interpretation of the selectivity terms (inverse mills ratio coefficients), it is recommended to adopt a plausible instrument(s) in the outcome equation for more robust identification (Deb and Trivedi 2006). To achieve this, we are required to have a selection instrument that is correlated with the decision to consume crickets but does not have a direct effect on the actual quantities of cricket sold (i.e. the effect of the instrumental variable on the outcome variable should only be through the decision to consume crickets). Following insights from previous studies on what makes a valid instrument in the context of our study and subject to passing falsification tests^6^ (Appendix 3) that validates usability, we included entomophagy awareness^7^ as an instrument (Christensen et al. 2006; Di Falcao et al. 2011; Manda et al. 2016; Midingoyi et al. 2019).

To estimate the average effect of consumption on market supply, we derive the expected actual and counterfactual outcomes using Eq. (6a, 6b). The expected actual outcome that is observed from the data is computed for cricket consumers, as follows:

9a $$E\left( {Y_{1i} {|}C = 1} \right) = \beta_{1} X_{i} + \sigma_{1} \lambda_{i}$$

The expected value of the counterfactual outcome for non-cricket consumption is given as follows:

9b $$E\left( {Y_{2i} {|}C = 1} \right) = \beta_{2} X_{i} + \sigma_{2} \lambda_{i}$$

where the $\beta_{2}$ and the $\sigma_{2}$ are the regression coefficients obtained from the outcome equation for regime 2 (non-cricket consumers).

The average cricket consumption effect on the treated group (ATT) and untreated group on market supply is computed as:

10 $$ATT = E\left( {Y_{1i} {|}C = 1} \right) - E\left( {Y_{2i} {|}C = 1} \right) = X_{i} \left( {\beta_{1} - \beta_{2} } \right) + \lambda_{i} \left( {\sigma_{1} - \sigma_{2} } \right)$$

11 $$ATU = E\left( {Y_{1i} {|}C = 0} \right) - E\left( {Y_{2i} {|}C = 0} \right) = X_{i} \left( {\beta_{1} - \beta_{2} } \right) + \lambda_{i} \left( {\sigma_{1} - \sigma_{2} } \right)$$

In Eq. (10) the terms $X_{i} \left( {\beta_{1} - \beta_{2} } \right)$ and $\lambda_{i} \left( {\sigma_{1} - \sigma_{2} } \right)$ denote the contribution of observed and unobserved heterogeneities to ATT respectively.

Finally, we estimate the transitional heterogeneity effects (TH), which is whether the effect is larger or smaller for the households that practice cricket consumption or for the households that did not practice cricket consumption in the counterfactual case that they did practice cricket consumption [i.e. the difference between Eqs. (10) and (11)]. A clear illustration of the computation of ATT and ATU and TH is presented in Table 1.

Given the assumption that the error terms have a trivariate normal distribution (Eq. 7), the ESR model can be efficiently estimated by the Full Information Maximum Likelihood method (Lee and Trost 1978; Lokshin and Sajaia 2004). The method yields consistent standard errors by simultaneously estimating the selection (probit) and the outcome equations.

## Results and discussion

### Summary statistics

Table 2 presents the summary statistics for the variables used in the empirical analysis. The choice of the covariates was based on a review of existing literature on the constraints and economic potential of insects and their contribution to food and nutrition (e.g., Dzerefos and Witkowski 2014; Halloran et al. 2015; Kelemu et al. 2015; Han et al. 2017; Ebenebe et al. 2017; Bermúdez-Serrano 2020; Cadinu et al. 2020; Babarinde et al. 2020), as well as the study context. In the summary statistics, we present a comparison of the means of the covariates by cricket rearing adoption status after exposure to training.

**Table 2:** Summary statistics of the survey sample from Western Kenya

	[1] Adopters (n = 139)		[2] Non-adopters (n = 162)		[3] Pooled (n = 301)		[1–2]
	Mean	Std.dev	Mean	Std.dev	Mean	Std.dev	t-test
Training sources (%): Local public universities					15.61	36.36	
NGOs					32.56	46.94	
Fellow farmers					51.83	50.04	
Production starter kit provided (% Yes)					28.57	45.25	
Amount of cricket harvested (Kgs)	3.09	5.44					
Amount of cricket sold (Kgs)	2.55	10.04					
Gender HH (1 = Male)	57.55	49.60	62.96	48.44	60.47	48.97	0.96
Age of HH in years	45.84	13.53	49.38	13.27	47.59	13.48	− 1.87*
Formal education of HH in years	9.53	3.45	9.26	3.37	9.39	3.40	0.69
Household size (count)	4.90	2.15	4.97	2.04	4.94	2.09	0.29
Land Size in acres	1.72	1.95	1.75	1.81	1.74	1.87	− 0.10
Total annual household income in KES^a^	255,25.20	1099123.00	88258.02	172075.20	162519.60	761209.70	1.91*
Distance to main road in walking minutes	10.35	10.90	12.06	13.87	11.27	12.59	− 1.18
Distance to main market in walking minutes	48.92	56.74	55.75	42.64	52.53	49.74	− 1.19
Cricket consumption (%Yes)	67.62	46.60	37.65	48.60	51.50	50.06	5.42***
Access to Extension (% Yes)	35.25	47.95	33.95	47.50	34.55	47.63	0.24
Group Membership (% Yes)	86.33	34.48	77.16	42.11	81.40	38.98	2.05**
Access to credit (% Yes)	30.21	46.09	28.40	45.23	53.01	50.06	0.35

Quantities of crickets harvested and sold are reported in kilograms (Kgs) in a 3-weeks cycle; HH-Household head; KES-Kenyan Shilling; Level of significance: *10%, **5%, ***1%

^a^Exchange rate at the time of the survey: $1 (USD) = Ksh. 104

About 26%, 35%, and 39% of the sampled farmers received training on cricket farming from a public university, NGOs, and fellow farmers respectively. This finding underscores the important role of rural networks in generating knowledge spillovers (positive) necessary for the adoption of new farm enterprises and technologies (Pratiwi and Suzuki 2020). We note that 46% of the respondents adopted cricket farming after receiving training. Notably, 58% of the adopters and 63% of the non-adopters are male. The adopters harvest and sell approximately 3.1 kg and 2.6 kg, respectively, per harvest cycle.^8^ Expectedly, 68% of the adopters report being cricket consumers, compared to 38% of the non-adopters. A comparison between adopters and non-adopters shows a significant statistical difference in consumption status. This finding implies that teaching potential cricket farmers how and why they should consume crickets first would likely lead to higher adoption of cricket farming as a source of livelihood.

The average age of the farmers was 48 years with non-adopters older (49 years) than adopters (46 years) with a marginal statistical difference. Years of formal education and household size were averagely 9 and 5 years, respectively, and no significant statistical difference was observed between adopters and non-adopters. The average statistically significant difference in annual income between adopters (KES. 255,256) and non-adopters (KES. 88,258) emphasizes the economic importance of cricket farming in transforming rural incomes.

With regard to access to institutional support services, 35% of both adopters and non-adopters reported having accessed credit. Interestingly, there was a significant difference (at 5% level) between the two groups with regards to membership in a rural group with about 86 and 77% positive responses among adopters and non-adopters, respectively. This finding is consistent with existing literature on the role of social networks and rural institutions in the adoption of new farming enterprises such as cricket farming (Bandiera and Rasul 2006; Kaufman et al. 2009; Binam et al. 2017; Weyori et al. 2018).

### Empirical results

#### Domestic cricket production determinants and constraints

In this section, we discuss the results of the Heckman two-step sample selection model presented in Table 3. The statistically significant inverse mills ratio confirms the presence of sample selectivity bias thus justifying the use of Heckman sample selection model. Further, the highly significant Wald test implies that the model fits our data well.

**Table 3:** Results of the probit and outcome equations of the Heckman two-step sample selection model of cricket farming

Independent variables	Probit		Outcome	
	dy/dx	SE	dy/dx	SE
Age of household head (log)	− 0.214	0.145	0.773*	0.467
Formal Education	− 0.008	0.013	− 0.071*	0.039
Household Size (log)	0.084	0.089	0.545**	0.260
Annual income (log)	0.038	0.039	0.312**	0.126
Distance to main road (log)	− 0.024***	0.007	− 0.053***	0.017
Credit access (1 = yes)	0.003	0.043	0.225*	0.117
Group membership (1 = yes)	0.220**	0.078	0.319	0.309
Insect consumption (1 = Yes)	0.362**	0.071		
Production starter kit provided (1 = yes)	0.526***	0.176		
Constant	2.051	3.598	− 1.957	2.308
Inverse mills ratio (λ)			− 0.548**	0.227
Number of observations	301			
Censored observations	92			
Uncensored observations	209			
Wald test χ^2^ (8)	39.89			
Probability χ^2^	0.000***			

dy/dx: marginal effects; SE: Standard error. The variables age, household size and annual income were logarithmically transformed. The dependent variable in the selection equation (probit) is = 1 if the respondent adopted cricket farming after receiving training and = 0 if otherwise. The dependent variable in the outcome equation is the cricket yield in kilograms for households that adopted cricket farming. Level of significance: *10%, **5%, ***1%

Results from the selection (probit) equation reveal that distance to main road, group membership, insect consumption, and provision of rearing equipment affects the probability of adoption of cricket farming after receiving training. Notably, holding all other factors constant, longer distance to the main road undermines adoption of cricket farming as it is associated with a reduction in the probability of adopting the enterprise after exposure to training by 2.4%. Previous studies have shown that poor rural road network could potentially limit timely access to inputs, technical support and financial resources necessary for agricultural production (Dorosh et al. 2010, 2012; Porter 2014). Additionally, longer distances to these services (remoteness) could translate to higher transaction costs (Chamberlin and Jayne 2013; Stifel and Minten 2017). Our finding is consistent with existing literature that remoteness in rural communities negatively affects adoption of new agricultural technologies (Staal et al. 2002; Olwande et al. 2009; Asfaw et al. 2011; Parvan 2011).

Membership to a rural group, practicing entomophagy, and provision of production starter kits after receiving training are associated with an increase in the probability of adopting cricket farming by 22%, 36%, and 53% respectively. Rural groups are a social capital forming and accumulation mechanism. As such, they facilitate access to key information that favor the uptake of new farming systems such as cricket farming (Weyori et al. 2018). Additionally, households that readily practice entomophagy are more willing to accept and integrate cricket farming as a source of livelihood (Halloran et al. 2020). However, investing in domestic cricket farming implies being willing to incur some initial set-up costs. As a result, the provision of rearing equipment offsets these costs thus increasing adoption likelihood.

The results of the outcome equation show that cricket yields are positively associated with larger household sizes, age of the household heads, higher incomes, and access to credit. Cricket farming is a labor-intensive enterprise and since the household size is a proxy for household farm labor, bigger households may have the advantage to produce more than smaller households. The positive effect of age is consistent with existing literature that entomophagy is mostly practiced by relatively older consumers (van Huis et al. 2013a, b; Vantomme 2015). This implies that older cricket farmers would invest more financial resources and time resulting in higher yields. Similarly, higher incomes and access to credit provide cricket farming households the means to meet the set-up costs, purchase the required equipment, and in some cases hire extra labor. However, the inclusion of income as a sum of both on-farm and off-farm incomes in our model makes our results contrary to those by Halloran et al. (2020) that willingness to participate in cricket farming decrease with higher household off-farm incomes.

The negative correlation of more years of schooling on cricket production suggest that relatively educated cricket farmers concentrate most of their resources (both financial and time) on other farm or non-farm activities. Finally, the negative association between inaccessibility to roads (remoteness) with cricket yields corroborates evidence from existing studies that poor road network in rural areas limits farmers’ access to both factor and output markets thus undermining farmers’ full production potential (Dorosh et al. 2012; Porter 2014).

#### Effects of training sources on cricket production

In this section, we discuss the results of METE model. However, our focus is on the second stage of the METE model which estimates the effects of the training sources on the natural log of quantity of crickets harvested in kilograms. The model estimates are presented in Table 4. The first stage mixed multinomial logit estimates are presented in Appendix 1. The base category in our model is training from fellow farmers which allows us to restrict our analysis to the two formal institutional training sources (NGOs and Public Universities). The plausible intuition behind this is that access to training from other farmers is more of a knowledge spillover effect due to strong social networks in farming communities. Our intuition is also supported by findings from the summary statistics that rural group membership rate was significantly higher among adopters than non-adopters.

**Table 4:** Multinomial endogenous treatment effects (METE) regression estimates

Training source	Net cricket yield in kilograms	
		% Change
NGOs	0.554*** (0.016)	55.4
Public Universities	0.708*** (0.015)	70.8
*Selection terms *(*λ*)		
λ _NGOs	0.256*** (0.004)	
λ _Public Universities	− 0.607*** (0.002)	
Lnsigma	− 4.222*** (0.139)	
Controls	Yes	
N	301	

The baseline is cricket farmers that received training from other farmers; Robust standard errors in parentheses; Level of significance: *10%, **5%, ***1%. Full model estimates presented in Appendix 2

The estimates from selection terms show evidence of negative selection bias implying that unobserved factors that explain participation in training from either of the sources are correlated with cricket yields. After controlling for unobservable heterogeneity, our regression estimates show that the two institutional sources of training: Local public Universities and NGOs, had positive significant effects on the cricket yield. Specifically, 55.4% and 70.8% cricket yield gains can be attributed to access to training from NGOs and public universities respectively. These increments in production translate to approximately 1.7 Kgs and 2.2 Kgs per harvest (3–4 week cycle) respectively. The differential effects could be attributed to differences in skill-set endowments and specialization among trainers from the two sources of training, although this assessment is beyond the scope of the current study.

#### Determinants and impacts of cricket consumption on market supply

The estimates of the first stage of the ESR model are presented in Table 5.

**Table 5:** Endogenous switching regression first stage estimates

Variables	Consumpt (1/0)		Cricket market supply in Kgs			
			Consumers (n = 94)		Non-consumers (n = 45)	
	Coef	SE	coef	SE	coef	SE
Age of head (log)	0.344	0.545	− 0.511	0.439	0.575	0.426
Gender of head (1 = male)	0.353	0.325	− 0.117	0.228	− 0.108	0.259
Formal Education	0.024	0.053	0.045	0.042	0.075*	0.045
Household size (log)	0.247	0.316	0.170	0.226	− 0.389	0.276
Annual income (log)	0.070	0.119	0.122	0.090	− 0.002	0.089
Distance to road	− 0.357**	0.143	0.106	0.130	− 0.338***	0.113
Distance to main mkt	− 0.007*	0.004	0.000	0.001	− 0.001	0.004
Access credit	− 0.208	0.355	− 0.329	0.244	− 0.006	0.288
Nutrition perception	0.864***	0.292	− 0.072	0.137	0.179	0.285
Operating mode^a^	0.305	0.223	0.375**	0.166	0.059	0.179
Group membership	0.598	0.477				
Entomophagy awareness	1.612***	0.323				
Cons	− 8.186***	2.771	0.346	1.821	− 2.065	2.303
$\sigma_{1}$, $\sigma_{2}$			0.131	0.431	− 0.604*	0.331
Sigma1, Sigma2			0.158**	0.074	− 0.298**	0.119
Log-likelihood	− 243.21					
Wald test: *x*^2^ (10)	20.34**					
LR test of independence *x*^*2*^(1)	3.90**					

N = 139: The cricket consumption equation, which represents the determinants of cricket entomophagy/consumption is presented in column 2 while the determinants of market supply for consumers and non-consumers are presented in columns 3 and 4; Estimation is restricted to households who adopted cricket farming after training

^a^Operating mode of cricket enterprise: 1 = with partner(s) or as a group; 0 = individually. Level of significance: *10%, **5%, ***1%

The statistically significant likelihood ratio test of independence of the selection and outcome equations indicates that there is a positive correlation between cricket consumption and market supply. This implies the presence of endogeneity problem hence justifying the use of ESR model. Additionally, the Wald test is statistically significant indicating the goodness of fit of our ESR model.

First stage ESR estimates show that determinants of cricket consumption are distance to the main road, distance to the main market, nutritional perception, and entomophagy awareness. Both distances to main road and the market had negative effects on the decision to consume crickets. This is probably because access to roads and shorter distances to markets may facilitate access to information. As a result, households that are favored by this proximity may be relatively more informed on alternative food sources compared to their remote counterparts. Besides, existing literature points out that good road networks and market accessibility in rural areas improve household decision making and economic outcomes due to increased access to agricultural information services (Jacoby 2000; Migose et al. 2018; Kiprono and Matsumoto 2018; Gebresilasse 2018; Morgan 2019).

Farmers’ awareness of crickets as source of food and perceing them as nutritional had positive effects on consumption decisions. These findings are consistent with current literature on drivers of insect consumption. For instance, Schouteten et al. (2016) observed that increasing acceptance of insects as an alternative food group is mainly attributed to consumer awareness of the nutritional benefits associated with insects. Other recent studies have also shown that consumer knowledge, which informs awareness, influences willingness to consume insect-based foods, willingness to use insect-based feeds for livestock, and perceptions as a protein source (Piha et al. 2018; Kim et al. 2019; Okello et al. 2021).

With regard to determinants of market supply, the mode of operating cricket farming affected market supply among cricket consumers. As such, operating cricket farming/enterprise with partners or as a group with other farmers increases the quantities sold among the cricket-consuming households. This could be attributed to efficiencies resulting from labor pooling and diverse skill-sets when operating with partners as cricket farming is labor-intensive (Hellin and Meijer 2009; Markelova and Mwangi 2010; Shiferaw et al. 2011; Aku et al. 2018). Additionally, it could be relatively easier to overcome arising financial constraints when operating with peers as opposed to rearing crickets on your own (Shiferaw et al. 2011).

Years on formal education and distance to the main road had positive and negative effects respectively on market supply among non-cricket consuming households. This implies that holding all other factors constant, highly educated non-consuming households supply more to the market perhaps because they access relevant market information (Fan and Salas Garcia 2018). However, all other factors held constant, their market supply diminishes with remoteness as longer distances to the main road reduce their ability to access the market.

The estimated coefficient of correlation between the cricket consumption equation and the market supply function (sigma) is significantly different from zero. The results suggest that both observed and unobserved factors influence market supply gains (welfare outcomes) given the consumption decision. The significance of the coefficient of correlation between the consumption equation and the outcome equation indicates that self-selection occurred in the decision to accept and consume crickets as an alternative food/protein group.

The estimates for the average treatments effects (ATT and ATU) and the heterogeneity effect (HE) are presented in Table 6.

**Table 6:** Endogenous switching regression treamemt effects estimates

Consumption status	Outcome	Treatment effects
Consumers	$ATT = E\left( {Y_{1i} {|}C = 1} \right) - E\left( {Y_{2i} {|}C = 1} \right)$	0.205(0.097) **
Non-consumers	$ATU = E\left( {Y_{1i} {|}C = 0} \right) - E\left( {Y_{2i} {|}C = 0} \right)$	0.295(0.125) **
Heterogeneity effects	$TH = ATT - ATU$	− 0.09 (0.114)

Standard errors in parentheses; Level of significance: *10%, **5%, ***1%

The results reveal that cricket consumption (entomophagy) significantly increased market supply and has the potential to increase that of non-consuming producing households. Interestingly, the results in Table 6 show that the potential effect on non-consuming households is greater than the actual effects on those currently practicing entomophagy. Specifically, practicing cricket consumption increased market supply by 20.5% (ATT). Similarly, the effect of cricket consumption (entomophagy) on the market supply of non-consuming farmers (ATU) is 29.5%. This implies that non-consuming households/farmers’ market supply would increase by 29.5% should they switch from non-consumption to consumption status. Lastly, we find no evidence (i.e.insignificant negative heterogeneity effects) that the effects are smaller in cricket-consuming households as compared to non-consuming households.

## Conclusions and policy implications

In this study, we analyzed socio-economic and institutional factors that would sustain the adoption of edible cricket farming and estimated the effects of institutional training and cricket consumption on production and market supply. Firstly, in assessing what determines how much adopters produce, we hypothesized that this hurdle is dependent on the decision to take up cricket farming after receiving training. We applied the Heckman sample selection model to assess socio-economic and institutional factors that influence adoption and production. Secondly, we hypothesized that the training provider determines the ultimate quantities produced. To that effect, we estimated the effect of the training sources on the production of edible crickets using a multinomial endogenous treatment effects model. Lastly, we deploy an endogenous switching regression to estimate the effects of household consumption of crickets (entomophagy) on market supply.

Our findings confirm that after being exposed to training, cricket adoption is enhanced by shorter distance to the markets, membership in rural institutions, provision of rearing equipment (starter kits), and practicing entomophagy. The implication is that uptake of new cricket farming enterprises would require both national and county governments in Kenya in collaboration with development partners to implement policies that address road infrastructural challenges to the market. The policies should further encourage and promote rural institutional capacity building of community members on cricket farming technologies for food and feed. Lastly, sensitization and awareness creation on the nutritional and health benefits of entomophagy to households and economic wellbeing of youth and women is crucial.

Cricket yield is significantly higher for adopters that received technical training from various institutions. This implies that future programs should take a collaborative approach in providing specialized training and technical backstopping to assist cricket farmers in overcoming adoption barriers. Research and development partners should endeavor to provide support through the provision of affordable production equipment, entrepreneurial skills, financial support, value addition, professional training and market access.

Our results further confirm an effect of cricket consumption on market supply. Thus, commercial cricket mass production and market demand would readily be achieved if frequent sensitization campaigns on cricket consumption are widely carried out to encourage adoption, acceptability, and practices of entomophagy in the various communities and beyond. Nevertheless, despite the aforementioned insights and policy implications, this study has some limitations that can be addressed by future research. Firstly, the current study focuses on the cricket enterprise from a supply-side only. Secondly, this study does not look into the cricket farmers’ disadoption behavior and lastly the study relies on cross-section data which has empirical limitations in estimating causal effects. Future studies should therefore address these limitations by assessing the cricket demand drivers, farmers’ disadoption behavior and endeavor to overcome data and study design limitations to analyze the dynamics of adoption, demand and supply as well as causal effects of institutional and behavioral interventions on consumption and yields.

## Data Availability

The data is available upon reasonable request.

## Appendices

### Appendix 1

See Table 7.

**Table 7:** Mixed multinomial logit estimates on determinants of training source access

	NGOs	Public University
Gender head (1 = Male)	− 0.209 (0.727)	0.256 (0.577)
Age	− 0.017 (0.029)	− 0.058** (0.028)
Education	− 0.033 (0.127)	− 0.085 (0.107)
Household size	0.130 (0.165)	0.042 (0.142)
Annual income (log)	− 0.072 (0.216)	− 0.144 (0.232)
Distance to market (log)	− 0.449 (0.318)	− 0.083 (0.277)
Access credit	− 0.276 (0.630)	0.663 (0.578)
Consume crickets	0.188 (0.824)	− 2.104*** (0.711)
Rural group membership	2.379 (1.612)	0.426 (0.961)
Location	4.261*** (1.160)	1.902*** (0.685)
_cons	− 2.148 (3.807)	4.928 (3.096)

The baseline is cricket farmers that received training from other farmers; n = 301; Robust standard errors in parentheses; Level of significance: *10%, **5%, ***1%

We use a dummy for location as a proxy for proximity to training source as an instrument in training access equation. We presume that farmers close within Kisumu and Siaya are closer to training source since the one of the public university that offered training and the NGO are located in these two counties

### Appendix 2

See Table 8.

**Table 8:** Multinomial endogenous treatment effects model estimates

	Net cricket yield in kilograms	% change
NGOs	0.554 (0.016)	55.4
Public Universities	0.708 (0.015)	70.8
*Controls*		
Gender head (1 = Male)	0.031 (0.006)	
Age	0.003 (0.000)	
Education	− 0.022 (0.001)	
Household size	− 0.066 (0.002)	
Annual income (log)	0.086 (0.003)	
Distance to market (log)	0.022 (0.003)	
Access credit	− 0.140 (0.007)	
Consume crickets	0.688 (0.006)	
Rural group membership	− 0.192 (0.008)	
*Selection terms *(*λ*)		
* λ* _NGOs	− 0.018 (0.082)	
* λ* _Public universities	− 0.542 (0.117)	
lnsigma	− 4.222 (0.139)	

The baseline is cricket farmers that received training from other farmers; Robust standard errors in parentheses; Level of significance: *10%, **5%, ***1%

### Appendix 3

See Table 9.

**Table 9:** Falsification test for entomophagy awareness as an instrument for cricket consumption

	Consumption (1/0)	Cricket sales (Kgs)
Entomophagy awareness	0.524 (0.078)	0.323 (0.236)
Age (log)	0.150 (0.115)	0.365 (0.347)
Gender head (1 = Male)	0.098 (0.069)	− 0.049 (0.208)
Formal education	0.010 (0.011)	0.073 (0.034)
Annual income (log)	0.022 (0.023)	0.028 (0.070)
Distance to market (log)	− 0.028 (0.039)	− 0.009 (0.118)
Household size (log)	0.038 (0.067)	− 0.112 (0.203)
Group membership (1 = Yes)	0.089 (0.107)	0.030 (0.323)
Extension services access	− 0.058 (0.071)	0.002 (0.214)
Constant	− 0.762 (0.521)	− 1.543 (1.574)

Entomophagy awareness is only associated with the decision to eat crickets (at 1% level) but is not associated with cricket sales; Robust standard errors in parentheses

## Abbreviations

ATT: Average treatment effect on treated
ATU: Average treatment effect on untreated
CAPI: Computer-assisted personal interviews
ESR: Endogenous switching regression
FAO: Food and Agriculture Organization
HE: Heterogeneity effect
ICIPE: International Center of Insect Physiology and Ecology
KES: Kenya shilling
METE: Multinomial endogenous treatment effect
NGO: Non-governmental organization
OLS: Odinary least squares
SSA: Sub-Saharan Africa
TH: Transitional heterogeneity
UN: The United Nastions
USD: United States dollar

## Footnotes

1 On average, 1 USD = KES. 107 during the survey year, i.e. 2020. https://www.exchange-rates.org/exchange-rate-history/usd-kes-2020-04-30.

2 The Flying Food project was a collaboration between teaching and research public universities and non-governmental organizations (NGOs) to promote production and consumption of crickets rolled out in 2013 in Kisumu, Siaya, and Homabay counties of Kenya. The farmers would then sell most of their harvested cricket to the project partners and to other consumers locally. More information on Flying Food Project is available at: https://www.flyingfoodproject.com/our-project/.

3 We exclusively define cricket entomophagy in our study as the consumption of crickets for food and nutrition.

4 The Flying Food Project had two main training models: through partnerships with (1) Public Teaching and Research Universities based in the region and (2) Non-governmental organizations (NGOs). Locals were invited to voluntarily register and participate in these trainings. However, due to community knowledge spillovers, a third training model (i.e.from (3) Fellow farmers) emerged.

5 We apply a logarithmic transformation on the quantities of crickets sold to normalize the data on the dependent variable.

6 In our falsification test, we separately regress cricket consumption decision (binary) and quantities sold on the instrument. We present the estimates in Appendix 3.

7 We consider a respondent to have entomophagy awareness if he/she received training on how and why (importances) they should eat crickets. 53% of respondents reported to have coverered entomophagy aspects from their training sources.

8 Interview with a key informant revealed that it takes 3–4 weeks from rearing to harvesting mature crickets. We also established during the scoping study and discussion with one of the Flying Food project leaders that the project made arrangements for farmers to sell most of the harvested crickets to designated buyers. However, farmers could still sell locally to other buyers.
